# The moderating role of transformational leadership on the relationship between deviant workplace behaviors and employee turnover intentions in China

**DOI:** 10.3389/fpsyg.2022.1005055

**Published:** 2022-10-11

**Authors:** Linkai Qi, Naveed Iqbal Chaudhary, Kai Yao, Farhan Mirza, Rabia Khalid

**Affiliations:** ^1^Business School, Shanghai University of Engineering Sciences, Shanghai, China; ^2^Department of Business Administration, University of the Punjab (Gujranwala Campus), Lahore, Pakistan; ^3^Business School, Fudan University, Shanghai, China; ^4^Knowledge Unit of Systems and Technology (KUST) Department, University of Management and Technology (Sialkot Campus) Sialkot, Lahore, Pakistan

**Keywords:** deviant workplace behavior, workplace mistreatment, workplace bullying, workplace incivility, transformational leadership, turnover intention, China

## Abstract

This study aimed to analyze the effect of deviant workplace behaviors, such as mistreatment, bullying, and incivility on employee turnover intention and identify the transformational leadership role as a moderator. The data was collected through a survey questionnaire with the help of a purposive sampling technique. A total of 318 respondents’ data was gathered from university academic and general staff in China. The results were analyzed through SPSS and structural equation modeling structural equation modeling (SEM) software. The findings indicate that deviant workplace behavior, i.e., mistreatment, bullying, and incivility, significantly affect employee turnover intention. Moreover, a result shows that transformational leadership has a significant moderating role on the relationship between turnover intention and workplace bullying and incivility but was insignificant between turnover intention and workplace mistreatment. Lastly, implications and limitations were also discussed in this article.

## Introduction

Organizations face several deviant workplace behaviors and employee turnover issues in the present era, such as workplace mistreatment, bullying, and incivility (Ayoko et al., 2003; Sharma and Singh, 2016). Deviant workplace behavior has negative consequences on the workplace environment. Many researchers across the globe extensively research in the context of their respective countries (Ghosh, 2017; Fida et al., 2018; Shehawy, 2022). Prior research has examined the role of organizational and individual levels and provides in-depth consequences of deviant workplace behavior (Chen and Wang, 2019). However, deviant workplace behavior, such as workplace bullying, is not adequately addressed at a broader level and is also considered taboo (Salin, 2003; Iqbal et al., 2017). The studies on interpersonal aggression reflected that employees faced physical and psychological damage within their workplace (Baharom et al., 2017a,b). It has also been observed that employees are mistreated continuously, which impacts their working ability. A few studies try to capture violent behaviors and damages in passive, indirect obvious actions and counterproductive workplace behavior (Peng et al., 2016; Naseer et al., 2020). Workplace bullying is considered a type of psychological abuse around the globe within some organizations (Schyns, 2007).

Deviant workplace behavior, such as workplace mistreatment, bullying, and incivility, harms employees and organizations (Gill et al., 2011). Existing research has already been conducted on negative behavior worldwide in different study settings (Iqbal et al., 2017; Baharom et al., 2017b; Li et al., 2020). Looking into previous literature research on deviant workplace behavior and turnover intention is empirically less examined by the researchers in the context of China educational sector employees. Turnover intentions exist almost in all types of organizations because when employees know about the behavior of their colleagues and seniors, they leave the organization after some time (Coetzee and van Dyk, 2018; Sandhya and Sulphey, 2020). The employees leave the organization because of negative behaviors, such as workplace mistreatment, bullying, and incivility. Workplace mistreatment includes several forms of conduct including harassment, incivility, bullying, and verbal aggression. This kind of mistreatment comes from the top and middle-level management and does not allow their co-workers to work according to their plan. Thus, bullies and incivility exist in organizations, which force employees to leave their organizations (Han et al., 2016).

There are several negative impacts of deviant workplace behaviors, such as minimizing the organization’s productivity, lowering the employees’ morale, and the organization’s working environment (Aryati et al., 2018). A previous study argued that Chines institutions face critical issues of deviant workplace behavior and turnover intention (Lee, 2019; Qi et al., 2020). The problem of employee turnover has come to increase in more critical consideration, particularly in this new century (De Clercq et al., 2021). The most commonly studied turnover determinants are work fulfillment, aim to leave, authoritative responsibility, and quest for new employment conduct (Zahra et al., 2013). The study’s primary objective is to analyze the impact of deviant work behaviors on turnover intention. Because it is assumed that the presence of deviant work behaviors, such as workplace mistreatment, workplace bullying, and workplace incivility increased turnover intention. However, the impact of these factors can be undermined with the help of transformational leadership because it plays a moderating role in the turnover intention of the organization. Deviant workplace behaviors; mistreatment, bullying, and incivility cause damage to both organizations and individuals (Iqbal et al., 2017; Murad et al., 2021). It becomes the reason to down the morale of the organization’s employees and hurt their self-respect. It destroys the environment of an organization. A prior study on banking professionals facing workplace bullying, incivility, and organizational frustration; probably increases turnover intention (Chaudhry et al., 2017). Moreover, the study found that deviant workplace behavior such as bullying, counterproductive, and sabotage increases employee turnover intentions (Bashir et al., 2012).

This study tries to address this critical issue by sharing meaningful findings to enhance performance by reducing turnover. The present study provides a significant contribution and tries to fill the following research gaps regarding the impact of workplace bullying, incivility, and mistreatment on turnover intention. Moreover, this study advances the literature on deviant workplace behaviors and turnover intention, as well as moderating the role of transformational leadership. This research also supports the two relevant theories, social exchange theory and psychological contract theory, to justify the research model theoretically and effectively manage the working environment. Lastly, the present study also recommends effective strategies and information that could help to overcome the critical issue of organizations’ turnover intention.

## Literature review and theoretical framework

Numerous scholars used different underpinning theories to justify the relationship between deviant work behavior and employee turnover intention, for example, social learning theory, theory of organization equilibrium, social exchange theory, equity theory, expectancy theory, human capital theory, psychological contract theory, and resource-based. However, to justify the proposed research model, this study led under the theoretical support of the two most relevant theories: social exchange theory and psychological contract theory. The social exchange theory is a sociological concept that applies to interpersonal interaction to understand relationship dynamics. The social exchange theory is traced back to 1958, in “social behavior as exchange,” written by American sociologist George Homans. He builds a framework based on the combination of basic economics and behaviorism. According to social exchange theory, all interactions between people may be traced back to a fundamental need to trade goods and services. People always consider a relationship’s potential benefits and drawbacks, and when workers feel unsafe maintaining connections, they often choose to end them (Cook et al., 2013). The social exchange theory is based on the concept that a relationship between people is developed on cost–benefit analysis. Therefore, the context of social exchange theory helps to understand deviant work behavior (Henle, 2005; Chernyak-Hai and Tziner, 2014).

Several studies have been done over the past few decades on what causes employees to leave their jobs voluntarily (Bani-Melhem et al., 2020; Yang et al., 2020). Turnover intention results from non-respect of explicitly or implicitly agreed on rules by colleagues or organization management. It reflects that those employees voluntarily quit the organization in case of a breach of the agreement (Shah et al., 2020). The social exchange theory is unique because it does not view relationships based on emotional metrics but is based on logic to determine the balance between a relationship. Social exchange theory explains the social behavior of two parties when they interact. Similarly, the social exchange theory is relevant to this research perspective because it examines the interaction of employees with their colleagues, seniors, and employers (Ishak and Bohari, 2016). Therefore, an employee decides turnover intention based on the cost–benefit analysis, and employees and employers suffer significantly from high staff turnover rates.

Employee turnover is costly for businesses in many ways. There are obvious ways that these expenses are incurred (recruitment, selection, and training), but there are also less obvious ways (in the form of lost knowledge and reduced productivity; Biron and Boon, 2013; Hussain et al., 2020). Employee turnover has several complicated factors that are currently poorly understood. Researchers have focused on modifiable risk factors to understand employee turnover causes in the hopes that addressing these causes may lead to reductions in planned and actual turnover (Guan et al., 2014). Employee performance has been a significant concern for many businesses since the 1980s. However, despite many studies, the link between work productivity and employee turnover remains unclear to avoid detection or capture (Zimmerman and Darnold, 2009; Khan et al., 2021). This research established a target-similarity model based on the premise that social exchange connections may be targeted toward various social entities. Employees are required to be able to establish and maintain positive relationships with the organization throughout their work (Caillier, 2021). Employees who feel disrespected at work are less invested, have a higher feeling of withdrawal (including a higher likelihood of quitting), and engage in deviant behavior at work.

Moreover, due to many workplace relations, employees just decided to leave that relationship by leaving the organization (Ali et al., 2022). Similarly, the psychological contract theory explains employee hold about the obligation of the mutual agreement between the employee and the organization (DelCampo, 2007). Moreover, the individual believes that the organization fulfills its obligations and promises (Robinson and Rousseau, 1994). Despite this, no research has systematically examined how transformational leadership and employee turnover intention influence deviant workplace behavior and organization-based social exchange processes.

### Deviant workplace behavior, workplace mistreatment, and turnover intention

Prior studies are conducted on deviant workplace behavior that connects workplace mistreatment to burnout; thus, turnover intentions become the result of burnout (Laschinger and Fida, 2014; Laeeque et al., 2018; Najam et al., 2018). Moreover, researches reflect a significant relationship between workplace mistreatment and turnover intentions in cross-sectional studies (Blackstock et al., 2015; Namin et al., 2021). According to previous scholars, some effects of mistreatment are identified, such as anxiety, depression, and, ultimately, intent to leave the organization (Laschinger and Fida, 2014; Wang et al., 2021). Nguyen and Stinglhamber (2020) explored the role of workplace mistreatment from abusive supervision, co-worker incivility, customer incivility, and organizational dehumanization to predict different outcomes such as turnover intention, job satisfaction, and emotional exhaustion. The study’s overall findings show that workplace mistreatment is closely relevant to the factors of turnover intentions and job satisfaction. When organizations mistreat employees, they tend to leave the organization and vice versa. Yang et al. (2014) contributed a significant piece of knowledge about workplace mistreatment. This research study examines the workplace mistreatment climate and its potential outcomes. The workplace mistreatment climate is taken as shared and individual perceptions of policies, practices, and procedures that discourage interpersonal mistreatment.

Furthermore, Yang et al. (2014) conducted a study using meta-analysis. They found a significant correlation between workplace mistreatment and organizational outcomes, such as mistreatment reduction effort strains, job attitudes, and mistreatment exposure. Haq et al. (2021) measured that workplace mistreatment results are highly stable at the employee level. The findings of this study reflect that if organization policies, processes, and procedures reduce the mistreatment working environment, employees are less intended to have turnover intention. However, vice versa, if organizations do not implement healthy policies, procedures, and processes, employees leave the organization because of uncontrolled deviant workplace behavior or mistreatment. So, the previously available literature reflects a significant correlation between workplace mistreatment and turnover intention (Nguyen and Stinglhamber, 2020; Zhou et al., 2021). These studies show a direct relationship between workplace mistreatment and turnover intentions because when mistreatment reduces, the turnover intentions also reduce and vice versa. Thus, based on the above literature, the following hypothesis is derived.

*H1*: There is a positive relationship between workplace mistreatment and employee turnover intentions.

### Deviant workplace behavior, workplace bullying, and turnover intention

The second issue of deviant workplace behavior is workplace bullying. Workplace bullying can also be psychological violence (Razzaghian and Ghani, 2014; Coetzee and van Dyk, 2018). Workplace bullying is concerning for both organizations and individuals (Blackstock et al., 2015). There is extensive literature on workplace bullying, which further correlates with organizational, social, and psychological aspects (Houshmand et al., 2012; Ahmad and Kaleem, 2019). It is assumed, based on the observation that an extreme situation of workplace bullying leads to turnover intention. Glambek et al. (2014) stated that a distinctive way from work environment bullying to prohibition from work might be through turnover that the target may encounter the working conditions as so troublesome that he or she chooses to take off the work voluntarily. Workplace bullying has been distinguished as an antecedent of job disappointment, which has been identified as a predecessor to turnover intentions (Kang and Jeong, 2019). There is an indecisive link between absenteeism, turnover intentions, and actual turnover. The most critical part is the intention to disband the organization (Najam et al., 2018).

Razzaghian and Ghani (2014) conducted a study examining the impact of workplace bullying on the turnover intention of faculty members. This study is closely linked with our research model because it is also done on faculty members of an educational institute. This study argued that deviant behaviors, like bullying, are complex actions that are more severe than sexual harassment. Such bullying behaviors turn into a dysfunctional atmosphere, leading the employees to turnover intention when the organization does not systematically address it (Salin and Notelaers, 2017). This research study shows a strong positive relationship between bullying in the workplace and business intent. A systemic review on deviant workplace behavior cited (Appelbaum et al., 2006) and reported that “almost 1.7 million Americans and 11% British employees experience a different type of bullying, such as physical assault, on-verbal, threatening, intimidating, humiliating, sabotage, interference with the production and exploitation at the workplace.”

Therefore, workplace bullying and turnover intentions are widely researched from different perspectives. Coetzee and van Dyk (2018) contributed to this aspect by examining workplace bullying and turnover intention by taking work engagement as a potential mediator. De Cieri et al. (2019) reported that 42% of health workers and nurses experienced workplace bullying in the previous 12 months. Furthermore, findings stated that employees’ turnover intentions have expensive and negative consequences for organizations. Therefore, work engagement plays a partial mediating role in the impact of increased workplace bullying on increased turnover intention (Abbas et al., 2018). Moreover, this study used the social cognitive theory as theoretical support for their model (Coetzee and van Dyk, 2018). Hence, the following hypothesis is developed based on the literature findings mentioned above.

*H2*: There is a positive relationship between workplace bullying and turnover intentions.

### Deviant workplace behavior, workplace incivility, and turnover intention

Workplace incivility is also deviant workplace behavior like mistreatment and bullying. Incivility at the workplace also negatively impacts both organizations and employees (Huang and Lin, 2019; Tricahyadinata et al., 2020). It is essential to address the problem to enhance its productivity and performance. A systemic review on deviant workplace behavior by Baharom et al. (2017a) cited the findings of the study of Alias et al. (2013); 71% of respondents reported incivility at the workplace. Workplace incivility becomes part of the usual routine when incivility is allowed to continue, and no one takes notice, which lets others engage in the same behaviors (Oyeleye et al., 2013). Unfortunately, such behaviors are being ignored by leaders who are not trained enough to handle the situations smartly (Laschinger and Fida, 2014). Existing studies argued that employees facing incivility become exhausted and thus misbehave with their co-workers (Han et al., 2016; Chen and Wang, 2019). Rahim and Cosby (2016) findings showed that workplace incivility has a significant relationship with turnover intention, which is mediated by job burnout. The study also identifies that workplace incivility is negatively linked with job performance. The employees who experienced greater incivility have a greater turnover intention and lower job performance.

Furthermore, a prior study found that workplace incivility significantly influences employee turnover intentions (Manzoor et al., 2020). Workplace incivility is also researched by associating it with satisfaction and turnover intention (Chen and Wang, 2019). The essential purpose of this paper was to examine the relationship between workplace incivility, job satisfaction, and turnover intention. The factor of emotional intelligence is taken as moderating variable. The findings showed that workplace incivility is negatively linked with job satisfaction, but workplace incivility is positively associated with turnover intention. When workplace incivility increases, turnover intention increases, and job satisfaction decreases. The study also indicates that emotional intelligence plays a significant moderating role in these relationships. Thus, based on these findings extracted from the literature, a hypothesis is developed between workplace incivility and turnover intention.

*H3*: There is a positive relationship between workplace incivility on employee turnover intentions.

### Moderating role of transformational leadership and turnover intention

Transformational leadership is a kind of leadership approach that brings social systems and individuals (Siangchokyoo et al., 2020). One of the most researched types of leadership, transformational leadership has been examined extensively for its ability to boost productivity at all levels of a company (Eliyana and Ma’arif, 2019). The question at the foundation of the research on employee turnover is this: what motivates workers to quit their jobs.” Transformative leaders inspire their followers to achieve both ambitious and previously unimaginable goals (Buil et al., 2019). More dedicated and contented followers are another hallmark of a transformational leader.

Moreover, transformational leaders enable their followers and pay attention to their unique requirements and growth, therefore fostering the growth of their followers’ inherent leadership abilities (Islam et al., 2021). Ideally, it brings or causes a positive and valuable change in the social system and its followers. In some respects, transformational leadership may be seen as an extension of transactional leadership. When leaders and subordinates all interact with one another, it is referred to as transactional leadership because of the emphasis placed on facilitating this trade; the leader must first explain the conditions under which the other participants must act and the benefits they would get if they do so (Farahnak et al., 2020). However, leadership that brings about transformational change is of a higher order. By coaching, mentoring, and providing both challenge and support, transformational leaders may help their followers grow into capable leaders while encouraging people to commit to the organization’s or unit’s shared vision and goals (Bass and Riggio, 2005; Lai et al., 2020).

Transformational leadership is a procedure where pioneers and adherents help each other progress to a more elevated amount of assurance and inspiration (Singh et al., 2020). This leadership approach is significant in the context of an organization because it brings critical changes in associations and individuals’ lives (Peng et al., 2021). It enhances the qualities, brings desired changes, and motivates workers toward their goals (Bacha, 2014). They assemble individual and socially recognizable proof among supporters of the mission and objectives of the leaders and organization. Based on the characteristics of transformational leaders, it is taken as a moderating variable for this research study. It is assumed that transformational leadership can play a moderating role in the relationship of turnover intention with workplace mistreatment, bullying, and incivility. There is extensive literature available that also used transformational leadership as a moderating variable within their research model of examining the workplace environment (Miao and Cao, 2019; Phung et al., 2019). Howladar (2018) researched deviant behavior at the workplace with job performance by setting transformational leadership as a moderating variable. The study’s findings reflect that transformational leadership moderates the relationship between deviant workplace behavior and job performance.

Likewise, prior studies utilized the transformational leadership factor as a moderating variable between customers in a civility environment. Several studies appraised behaviors of formational leadership as moderators in the subsequent relationships (Iqbal et al., 2017; Baharom et al., 2017a), and the findings of these studies showed that transformational leadership plays moderating role in minimizing deviant work behavior. Katou et al. (2020) most recently evaluated the moderating effect of transformational leadership between self-worth and employees’ reactions. Findings show a significant role of transformational leadership in overcoming negative behaviors in the workplace. Consequently, the literature mentioned above reflects a significant relationship between transformational leadership and workplace mistreatment, bullying, and incivility. Therefore, based on the above review following hypotheses are formulated.

*H4*: Transformational leadership has a significant moderating role in the relationship between workplace mistreatment and turnover intentions.

*H5*: Transformational leadership has a significant moderating role in the relationship between workplace bullying and turnover intentions.

*H6*: Transformational leadership has a significant moderating role in the relationship between workplace incivility and turnover intentions.

### Theoretical framework

**Figure 1:**
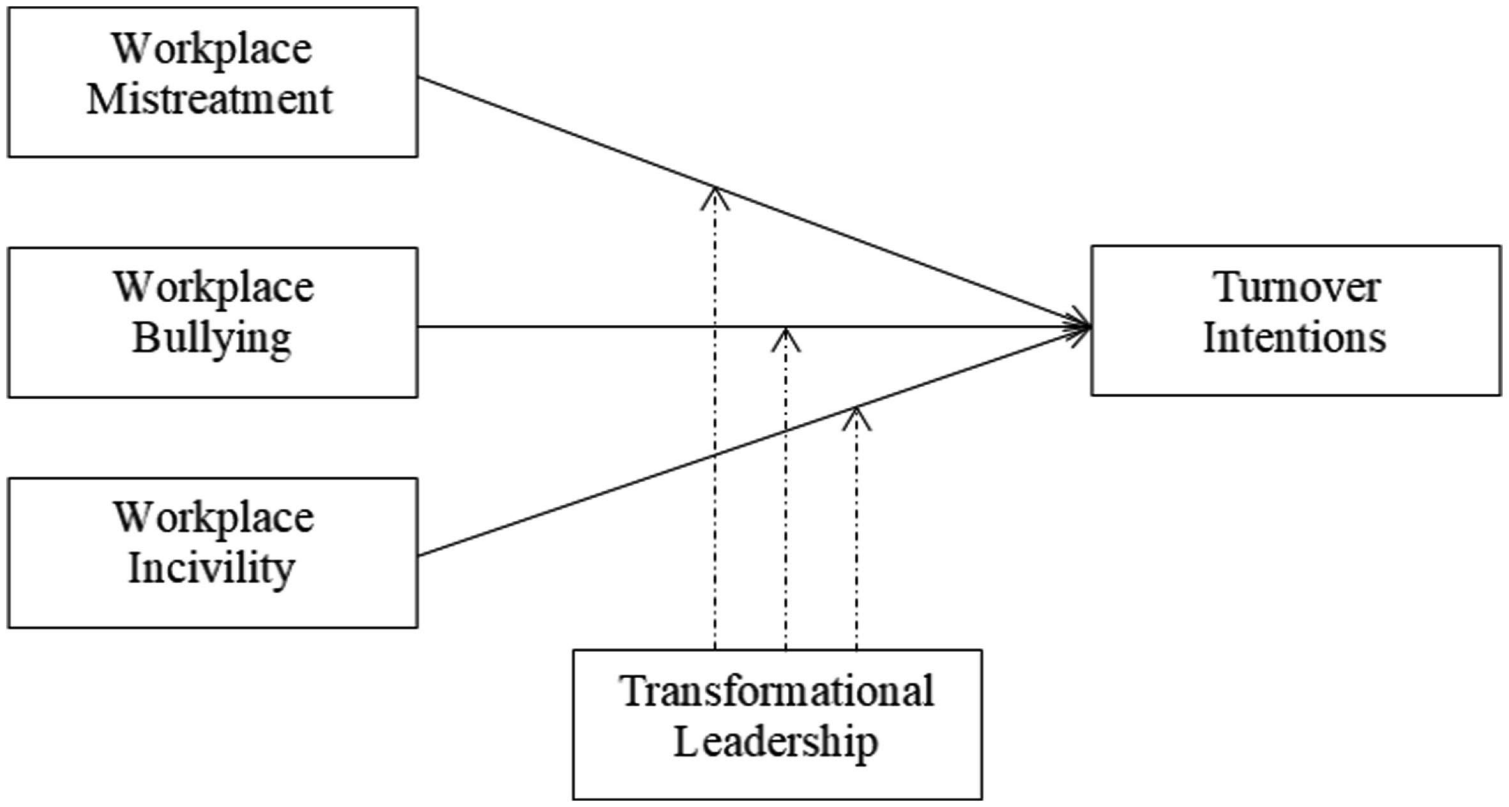
Conceptual framework.

Figure 1 presents the research model of the study. The dependent variable turnover intention is the outcome of the independent variables, workplace mistreatment, bullying, and incivility. Transformational leadership plays a moderating role in this relationship.

## Research design/methodology

### Sample and data collection

The nature of this study was exploratory and cross-sectional. This study’s population consisted of 10 large category universities in Shanghai, China. The data were gathered from academic and general staff employees of different departments of universities due to their workplace environment and turnover ratio. This study used a purposive sampling technique for sample determination because it provides a broader range of non-probability sampling opportunities for the whole population. The sample size was determined using the criteria suggested by Cochran et al. (1954).

Moreover, this study developed a survey-based questionnaire sent to respondents through email and other means. The online method was selected because the researcher could not visit the study area physically, and currently, there is a pandemic of COVID-19 and university staff is working from home. A total of 380 questionnaires were mailed to the finalized sample on their official email and social media accounts such as WeChat. The data collection took 6 weeks, and two reminder emails were sent every 2 weeks after the initial mail. Thus, 345 responses were generated from the original 380, entailing a response rate of 90.7 percent. These 345 responses were analyzed for missing and repeat values. The missing and repeated values were further eliminated from the data. Thus, the total usable number of responses was 318. The remaining 27 were discarded due to missing values and repeat responses.

### Measures

The questionnaire for this study is adapted from prior studies. After extracting the questionnaire from existing literature, we made some changes to it due to the context changes. Moreover, we converted the scale to a five-point Likert scale ranging from 1 strongly disagree to 5 strongly agree. A scale of eight items was adapted from the study by (Harlos and Axelrod, 2005) to measure workplace mistreatment. It has good reliability in previous literature, and a sample item is “I have been blamed for others’ mistakes.” This behavior was measured on five points Likert scale from “never to all ways.” A scale of 22 items adapted from the study by Einarsen et al. (2009) is used to measure workplace bullying. A sample item is “someone withholding information that affects your performance,” It ranges from “never to daily.” A scale from this study by Cortina et al. (2001) was adopted to measure workplace incivility. It had 10 items with good reliability in prior literature. A sample item “put you down or was condescending to you in some way” ranges from “once or twice a year to every day.”

Furthermore, the dependent variable is the turnover intention measured with the four items adapted from the study by Kelloway et al. (1999), whereas moderating variable transformational leadership, measured with the help of 12 items and a scale, was adapted from the study by Bass et al., (2003) from “not at all too frequently.” Furthermore, all scales are converted into numeric values for quantitative analysis, and each option is given a different number in the value option. For example, the question under workplace is treatment headings follow five items that start from “never to always.” Similarly, five items are used for the workplace bullying variable: never, now and then, monthly, weekly, and daily.

### Data analysis method

We used statistical packages for social sciences SPSS and analysis of moment structures AMOS for data analysis. The descriptive analysis, preliminary testing for missing values and outliers, and factor analysis were performed on SPSS. The confirmatory factor analysis and structural equation modeling procedures were performed in AMOS.

## Results

The demographic profile for this research shows that 318 respondents belong to China’s educational sector. Most of the respondents are male in terms of gender, and in the context of age, the highest percentage is noted from age 36–45. However, most of the respondents have done graduation, whereas very few have done a Ph.D. Following, Table 1 shows their details.

**Table 1:** Demographic information respondents’ profile.

		Frequency	Percent
Gender	Male	171	53.8
	Female	147	46.2
	Total	318	100.0
Age	25–35 year	99	31.1
	36–45 years	146	45.9
	46–55 years	73	23.0
	Total	318	100.0
Education	Undergraduates	46	14.5
	Graduation	140	44.0
	Masters	102	32.1
	Ph.D. or other	30	9.4
	Total	318	100.0

Table 1 shows that out of the total 318 respondents, 147 were females and 171 were males. There is low gender disparity in the sample showing that the educational sector of China does not have much gender discrimination. Most of the respondents were young, i.e., belonging to group ages 36–45 years, and 99 were between 25 and 35 years of age. Moreover, 73 respondents were between 46 and 55 years of age. Besides, 46 of the respondents had undergraduate degrees in terms of educational background, 140 had graduation degrees, 102 had degrees of Masters and 30 had higher degrees (Ph.D.) and other degrees.

### Descriptive statistics

The coefficients that explain and summarize a given data set are known as the set of descriptive statistics (George and Mallery, 2019). All these statistics describe the underlying features of the data and present insights into the sample and the measures. This study computed the valid responses, maximum and minimum range, mean, skewness, and standard deviation. Thus, the minimum and maximum values depict the range of the five-type Likert scale measurement scale used in the survey for this research.

The mean values represent the average of responses obtained for each item and show a tendency to locate around 3.3, showing that most responses were neutral. The measure of skewness values represents the amount of distortion or asymmetry in a data set (Groeneveld and Meeden, 1984). As shown in Table 2, the skewness values lie in the normal range between −1 to 1, showing that the data are accurately distributed and valid. Thus, the results of descriptive statistics prove that the data are valid for further testing.

**Table 2:** Descriptive statistics.

	N	Min	Mix	Mean	*SD*	Skew
WM	318	1.00	5.00	3.2072	0.87367	−0.396
WB	318	1.00	5.00	3.0214	0.98060	−0.089
WI	318	1.00	5.00	3.4396	1.01351	−0.643
TI	318	1.00	5.00	3.5157	1.08353	−0.448
TL	318	1.00	5.00	2.9248	1.00234	0.227

WM, Workplace mistreatment; WB, Workplace bullying; WI, Workplace incivility; TI, Turnover intentions; and TL, Transformational leadership.

### Sample adequacy and data sphericity tests

This study used the Kaiser Meyer Olkin (KMO) test to measure the adequacy of the sample. Suppose the value of the KMO indicator is found to be high, e.g., close to 1. In that case, it is an indication that performing a factor analysis is beneficial according to the data, while having values below 0.5 indicates that factor analysis will not reap many benefits (Chen and Fu, 2011).

Table 3 shows that the value of the KMO indicator is 0.931, which is significantly close to 1; therefore, the KMO test clears the data for factor analysis. The purpose of Bartlett’s test of sphericity is to ensure that the research variables are not overlapping and are unrelated by checking if the correlation matrix is an identity matrix or not.

**Table 3:** KMO and Bartlett’s test.

“Kaiser-Meyer-Olkin measure of sampling adequacy.”		0.931
“bartlett’s test of sphericity”	Approx. Chi-square	13277.122
	Df	1,540
	Sig.	0.000

#### Factor analysis

The rotated component matrix test was performed to assess the cross-loading of all the measurement constructs. According to previous researchers, the general rule is that the factor loadings of each factor individually need to be above the value of 0.50 (Hair et al., 1998, 2007). The component loadings of all scale items included in this research have been presented in Table 4. It can be seen that all the included factors have significant contributions, as all factor loadings are greater than 0.50. Moreover, the factors have not been observed to cross-load against each other, showing that the data have reliability. Therefore, the results confirm that the data are reliable and valid for further testing.

**Table 4:** Rotated component matrix.

	Component				
	1	2	3	4	5
WM1				0.755	
WM2				0.667	
WM3				0.693	
WM4				0.724	
WM5				0.732	
WM6				0.720	
WM7				0.738	
WM8				0.730	
WB1	0.781				
WB2	0.813				
WB3	0.777				
WB4	0.677				
WB5	0.684				
WB6	0.667				
WB7	0.660				
WB8	0.694				
WB9	0.773				
WB10	0.666				
WB11	0.713				
WB12	0.722				
WB13	0.728				
WB14	0.816				
WB15	0.838				
WB16	0.795				
WB17	0.755				
WB18	0.745				
WB19	0.737				
WB20	0.656				
WB21	0.677				
WB22	0.669				
WI1			0.865		
WI2			0.763		
W3			0.802		
WI4			0.732		
WI5			0.618		
WI6			0.793		
WI7			0.681		
WI8			0.622		
WI9			0.686		
WI10			0.690		
TI1					0.755
TI2					0.747
TI3					0.739
TI4					0.741
TL1		0.770			
TL2		0.632			
TL3		0.707			
TL4		0.598			
TL5		0.643			
TL6		0.707			
TL7		0.821			
TL8		0.751			
TL9		0.740			
TL10		0.737			
TL11		0.768			
TL12		0.758			

WM, Workplace mistreatment; WB, Workplace bullying; WI, Workplace incivility; TI, Turnover intentions; and TL, Transformational leadership.

#### Construct validity

For testing the validity of the constructs, two subtypes of construct validity are tested, i.e., convergent validity and discriminant validity which are both critical for ensuring that the constructs are all valid. The convergent validity is confirmed by showing that the repeated but differentiated measures for similar constructs provide the same results repeatedly (Cunningham et al., 2001). Convergent validity is evaluated based on three values, i.e., average variance extracted (AVE), composite reliability (CR), and maximum shared variance (MSV). AVE measures the amount of variance a construct captures compared to the amount of variance due to measurement error. CR is the internal consistency value in the scale items. The MSV is the maximum amount of variance in the constructs, and the values of MSV need to be lower than AVE. The thresholds for CR and AVE are 0.70 and 0.50, respectively (Henseler et al., 2015). Table 5 present the results of validity. It can be seen that the convergent validity is proven as the composite reliability values are above 0.70 for all variables, and the average value extracted is above 0.50 for each variable.

**Table 5:** Convergent and discriminant validity.

	Cronbach’s alpha	CR	Ave	MSV	WM	TI	TL	WI	WB
WM	0.902	0.901	0.533	0.245	0.730				
TI	0.875	0.875	0.636	0.244	0.494	0.797			
TL	0.934	0.928	0.525	0.245	0.495	0.490	0.724		
WI	0.910	0.908	0.499	0.135	0.299	0.368	0.345	0.706	
WB	0.965	0.963	0.545	0.241	0.409	0.491	0.386	0.329	0.738

WM, Workplace mistreatment; WB, Workplace bullying; WI, Workplace incivility; TI, Turnover intentions; and TL, Transformational leadership.

**Figure 2:**
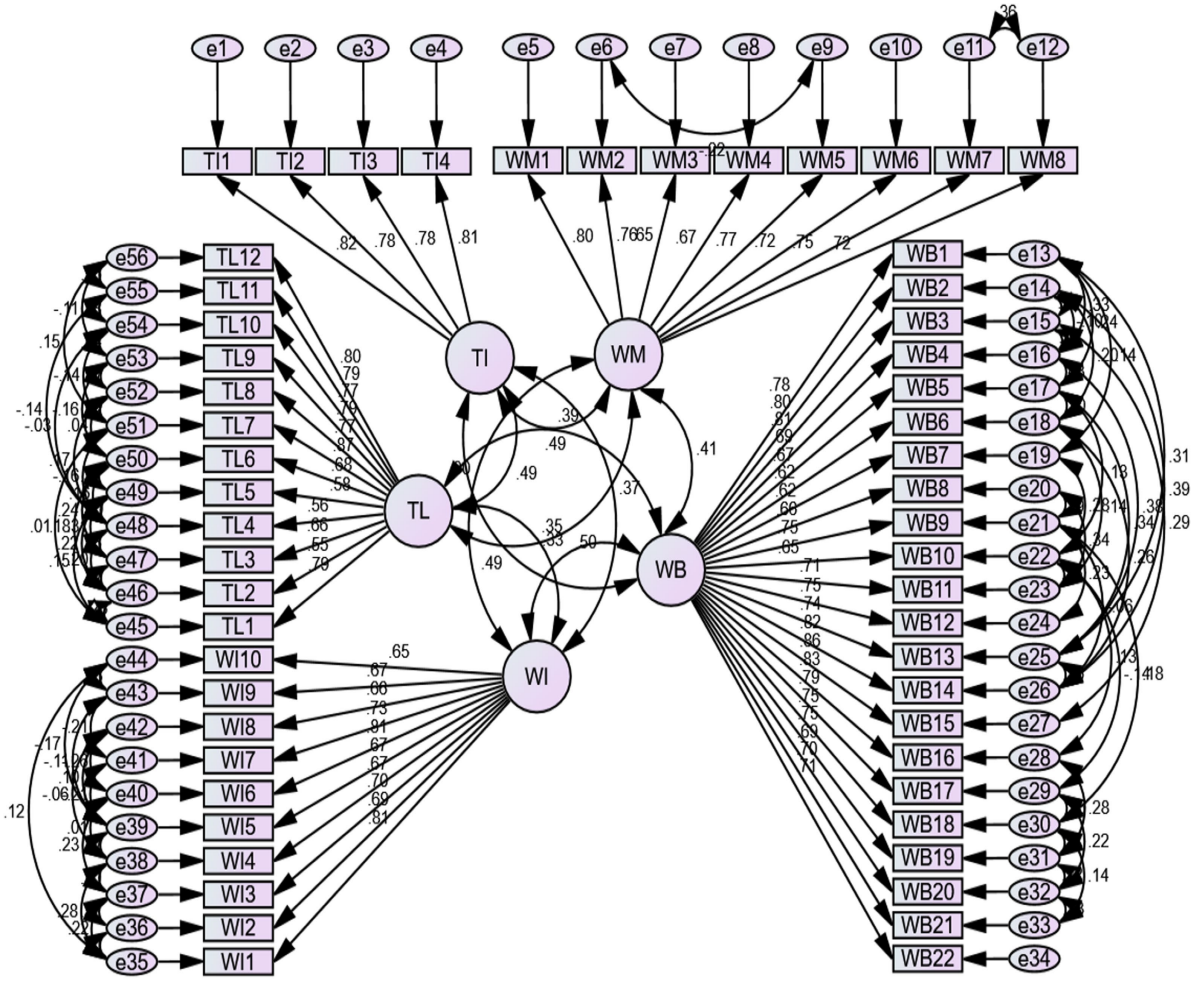
CFA analysis.

Table 6 shows the CFA results for this research, which confirms that the data are fit and the hypothetical model is fit before structural equation modeling. To achieve the excellent fit indices researcher applied the modification indices as suggested by Arbuckle and Wothke (1999). The current results show that CMIN is 1.665, which is less than 3, GFI is 0.802, which is more than 0.80, it is 0.927, which is more than 0.90, CFI is 0.926, which is more than 0.90, and finally, the value for RMSEA is.046 which is less than 0.08. All the results lie within their normal ranges, showing that the data are valid, suitable, and precise for further testing. Figure 2 indicates that all the variables load on each factor and are positively correlated.

**Table 6:** Model fit indices.

CFA indicators	Threshold value	Observed value
CMIN/DF	≤3	1.665
GFI	≥0.80	0.802
IF	≥0.90	0.927
CFI	≥0.90	0.926
RMS	≤0.08	0.046

### Structural equation modeling

Structural equation modeling (SEM) is a multivariate tool of statistical analysis used to combine the power and analysis of the factor and multiple regression analysis (Ullman and Bentler, 2012). SEM can incorporate several statistical frameworks as it is a flexible technique, and several researchers have applied this technique to test the hypotheses’ relationships between variables. Table 7 presents the regression weights of structural equation modeling.

**Table 7:** Regression weights.

Hypotheses	Regression			Estimate	SE.	CR.	P	Decision
H1	WM	→	TI	0.289	0.064	5.611	0.000	Accepted
H2	WB	→	TI	0.279	0.058	5.351	0.000	Accepted
H3	WI	→	TI	0.166	0.053	3.345	0.000	Accepted
	**Moderation Effects**			**Estimate**	**SE.**	**CR.**	**P**	**Decision**
H4	WM*TL	→	TI	−0.016	0.053	−0.310	0.757	Rejected
H5	WB*TL	→	TI	0.136	0.052	2.804	0.005	Accepted
H6	WI*TL	→	TI	0.163	0.050	3.316	0.000	Accepted

WM, Workplace mistreatment; WB, Workplace bullying; WI, Workplace incivility; TI, Turnover intentions; and TL, Transformational leadership.

**Figure 3:**
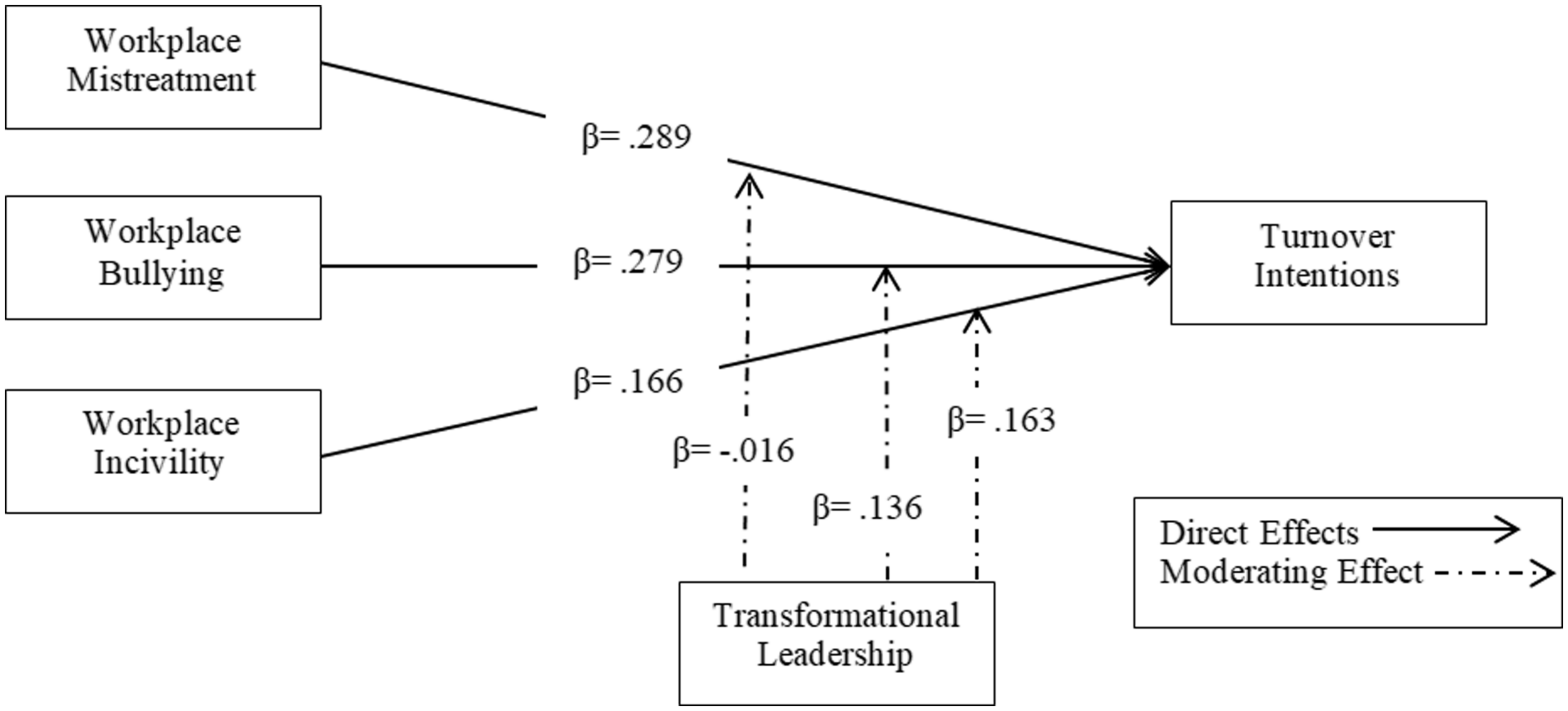
Structural equation modeling (SEM) model estimation.

Table 7 and Figure 3 show the results of hypotheses testing. The result indicates that workplace mistreatment has a positive and significant impact on turnover intention; as we can see, the coefficient against this statement is β = 0.289, which means that if one unit of workplace mistreatment increased, it would enhance a 28.9% turnover intention rate in organizations. So, we need to reduce the factor of mistreatment in the workplace to reduce turnover intention. Therefore, the first hypothesis of this study is accepted as the value of *p* is less than 0.05. The second hypothesis indicates that workplace bullying has a positive and significant impact on turnover intention. As a result, workplace bullying has a β = 0.279 positive impact on turnover intention because the value of *p* is.000, which is less than 0.05. So, if one unit of workplace bullying increases in the organization, it will bring a 27.9% increase in turnover intention. Therefore, the second purpose is supported. The third hypothesis is related to workplace incivility. The results show that workplace incivility has a β = 0.166 positive impact on turnover intention, which is significant. Hence, the third hypothesis is also accepted.

Furthermore, H4, H5, and H6 hypotheses are related to the moderating effect of transformational leadership. Results indicate that transformational leadership has no significant moderating effect between workplace mistreatment and turnover intention. While in the fourth and fifth hypotheses, transformational leadership has a positive and significant moderating effect. Therefore, hypotheses H4 and H5 are accepted. Moreover, the lower and high moderating effects of transformational leadership are presented below.

**Figure 4:**
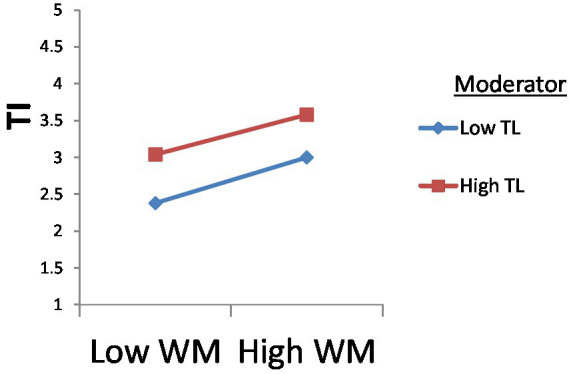
Moderating effect of TL between WM and TI.

Additionally, Figure 4 shows no significant moderating effect of transformational leadership in low or high perspectives.

**Figure 5:**
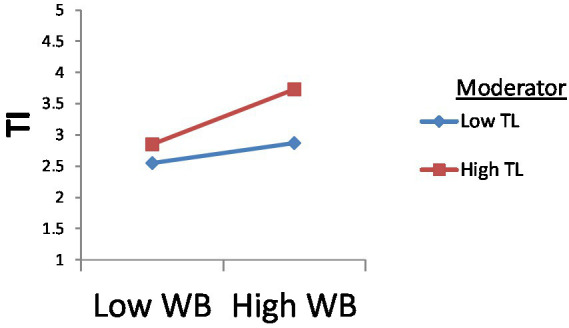
Moderating effect of TL between WB and TI.

Besides, Figure 5 indicates that transformational leadership has a significant moderating effect between workplace bullying and turnover intention. The lines on the graph show the interaction of transformational leadership between WB and TI, demonstrating that transformational leadership’s moderating effect on this relationship is positive and significant as a high line goes to the upper side of the graph.

**Figure 6:**
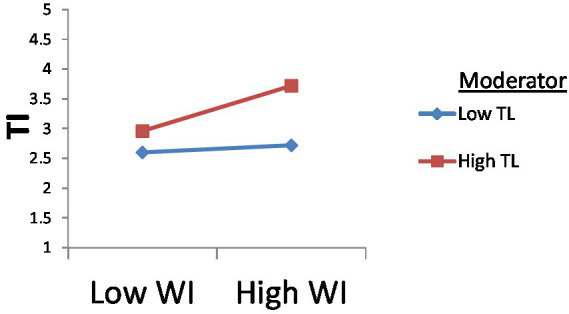
Moderating effect of TL between WI and TI.

Figure 6 shows the results of hypothesis H6, indicating that the relationship between workplace incivility and turnover intention is significantly moderated by transformational leadership. The interaction term of transformational leadership on the above graph shows that high transformational leadership has a high moderating effect on this relationship. The red line on the graph slightly goes on the upper side, which indicates the positive influence.

## Discussion

The previous literature shows that workplace mistreatment significantly impacts the employee and the organization’s performance (Nguyen and Stinglhamber, 2020). Workplace mistreatment is a social issue faced by employers, seniors, and co-workers at the workplace. Workplace mistreatment also impacts the health of employees who experience it, along with emotional and psychological injuries. This study’s finding is aligned with the assumption reflected by the prior studies (Yang et al., 2014; Zhou et al., 2021), who found similar results. Therefore, this study accepts the H1 that workplace mistreatment significantly impacts employee turnover intentions. The employees take the pressure of workplace mistreatment seriously and intend to leave the job or organization. Because the current job position and organization harm their physical and mental health and emotional damage, they are searching for a new job that is healthy and suitable for them (McCord et al., 2018). The findings of this research study identify workplace mistreatment as a meaningful predictor of leaving the job or organization. Thus, this discussion section achieves the first objective of this research study and answers the first question aligned with H1.

Discussing H2, the word bullying has various meanings that depict a wide assortment of encounters and circumstances. However, bullying is mainly defined according to the context of each country. The dismissive nature of bullying associated with essential to the concept of bullying might include being revealed to relentless insults or derogatory remarks, personal and even physical abuse (Hoel et al., 2002). Therefore, the second question is aligned with the existing studies of prior researchers who found that workplace bullying is positively associated with employee turnover intention (Blackstock et al., 2015; Coetzee and van Dyk, 2018). Furthermore, concerning H3, this study argues that workplace incivility is also a type of deviant behavior of low intensity with an undefined intent to harm the employees. The behaviors that come under workplace incivility are also defined as interpersonal mistreatment, implicit discourtesy, and disregard toward others that may lead to disorganization, violation of relationships, and abrasion of empathy. Previous studies, which are based on individual-level incivility, found that it can take many forms of incivility, such as reading emails or replying to text messages to humiliate others (Leiter et al., 2015; Chen and Wang, 2019). Therefore, the previously available literature reflects various outcomes of workplace incivility, but this study is only concerned with the outcome of employee turnover intention. When incivility is allowed to continue, no one notices, letting others engage in the same behavior. Unfortunately, leaders’ behaviors are ignored by leaders, resulting in employee turnover intentions (Laschinger and Fida, 2014). In other studies, researchers find out that employees facing incivility become exhausted and thus misbehave with their co-workers (Han et al., 2016; Manzoor et al., 2020).

Additionally, H4 explores the role of transformational leadership as a moderator on the relationship between workplace bullying and turnover intentions. Transformational leadership influences employees in achieving organizational goals and objectives, helps employees reduce deviant behavior, and enhances the working environment. Similarly, H5 findings show that transformational leadership is assumed to play a moderating role between workplace mistreatment and turnover intention. However, most literature used transformational leadership as a moderating variable within their research model to analyze the organization’s environment and negative interpersonal behaviors (Howladar et al., 2018; Islam et al., 2021). Interestingly, the present research study results are linked to the previous literature and the assumption that change in the relationship between bullying in the workplace and business intent plays a significant role. Therefore, we can accept the speculation that change leadership plays a buffering role between bullying in the workplace and employee business intentions.

Besides, H6 shows that transformational leadership is assumed to be a moderator for workplace mistreatment and bullying and is also assumed similarly for workplace incivility and turnover intention. Numerous studies reflect that transformational leadership helps achieve organizational goals and helps maintain a healthy environment within the organization (Arnold and Walsh, 2015; Miao and Cao, 2019). However, this research study analyzes that workplace mistreatment, bullying, and incivility explain most of the variations in employee turnover intention. By addressing these three factors, an organization may reduce the ratio of employee turnover intention to a significant level.

## Implications and limitations

This study has some practical and theoretical implications for educational institutes and policymakers, which is an uncovered area that needs research and exploration of deviant workplace behavior and turnover intention. Firstly, the study findings can be implied in the academic setting because it reflects that transformational leadership helps achieve goals and overcomes negative interpersonal relationships and the working environment. Secondly, the study can be helpful for organizations with the highest employee turnover ratio. Such an organization can examine its working environment critically by implementing strict policies and processes for those who harm other employees through mistreatment, bullying, and uncivil behavior. By addressing these three factors, they may reduce the turnover ratio to a significant level.

Moreover, this study is significant for the education sectors by implementing these factors to enhance the productivity and progress of organizations because there is a lack of studies related to these variables within the public sector organization. The organization must reshuffle the employee to another department or location based on the employee’s problem. In this way, the organization will face less employee turnover and keep the employee secure and sound from deviant behaviors. It is significant because it affects an employee’s psychological, social, and physical health.

Furthermore, the theoretical implications of the present research study can be followed. The study used psychological contract theory and social exchange theory as a theoretical justification for its research model. This aspect of the study can be used with a wide range of other factors for conducting similar research. It also helps to narrow the analysis process by increasing the probability of determining relevant factors. The findings of the present research study aid the utilization of an extensive theoretical framework, and the strengthened factors can be used for categories such as job attitudes and organizational factors. Therefore, the study research design can be replicated to examine work-related factors influencing employee turnover intention. However, few research studies in the educational context exist related to turnover intention. Therefore, this study enriches the available literature by providing meaningful and empirical outcomes.

This study is also subjected to offer some limitations and future research directions. The first fundamental limitation of the study is the limited sample size due to the short time. However, it is recommended to consider the large sample size and conduct longitudinal research for future studies. Another significant limitation is faced due to using a purposive sampling technique because, in covering the whole country, it is essential to set quotas according to their population and the number of educational institutes to get more accurate results. Therefore, it is recommended to use quota sampling when covering the highest population with distinction in the future. The data is collected through a questionnaire survey; other data collection tools can be used to strengthen the topic, such as interviews and group discussions. In addition, it will help bring more opinions and points related to the topic. Therefore, for future research, this research will recommend a comparative analysis of different provinces and federal or tribal areas to examine the difference or similarities of public section educational institutions’ processes, procedures, leadership roles, and workplace interpersonal relationship patterns. This comparative analysis of public sector educational institutes will give a deeper understanding of the organizations, employees’ interpersonal relationships, and cultural patterns.

## Conclusion

The study aims to identify the effect of deviant workplace behaviors such as workplace mistreatment, bullying, and incivility on employee turnover intention. Moreover, the moderating role of transformational leadership has been tested. The research study assumes that the organizations’ turnover intention increases due to deviant workplace behaviors, such as workplace mistreatment, bullying, and incivility. However, the impact of these factors can be overcome through transformational leadership because it plays a moderating role in turnover intention and managing the working environment effectively. This study applied the purposive sampling technique to collect the data. Finally, 318 respondents’ data was collected *via* a questionnaire from universities’ general and academic staff to test the study hypotheses. Findings obtained through structural equation modeling and confirmatory factor analysis state a significant positive impact of workplace mistreatment, bullying, and incivility on employee turnover intention. Transformational leadership significantly moderates the relationship between turnover intention and workplace bullying and incivility but is insignificant between turnover intention and workplace mistreatment. Lastly, current research indicates that the government in policymaking for the education sector implements the findings of these factors to enhance the productivity and progress of organizations.

## Data availability statement

The raw data supporting the conclusions of this article will be made available by the authors, without undue reservation.

## Ethics statement

The studies involving human participants were reviewed and approved by Shanghai University of Engineering Science, Shanghai, China. The patients/participants provided their written informed consent to participate in this study.

## Author contributions

LQ and NC proposed the research idea, analyzed the results, and wrote the manuscript. KY, FM, and RK carried out the methodology and extensively edited the manuscript. All authors contributed to the article and approved the submitted version.

## Conflict of interest

The authors declare that the research was conducted in the absence of any commercial or financial relationships that could be construed as a potential conflict of interest.

## Publisher’s note

All claims expressed in this article are solely those of the authors and do not necessarily represent those of their affiliated organizations, or those of the publisher, the editors and the reviewers. Any product that may be evaluated in this article, or claim that may be made by its manufacturer, is not guaranteed or endorsed by the publisher.
